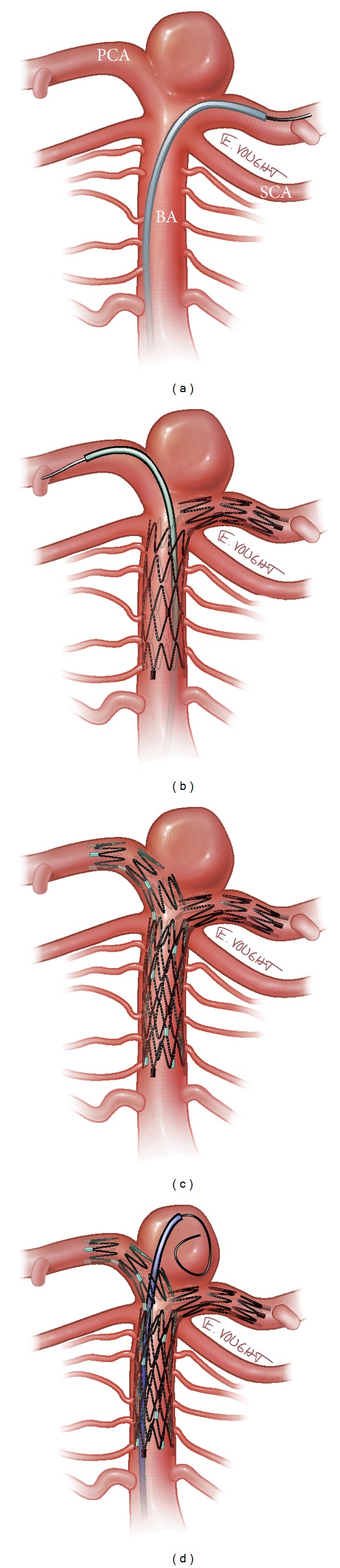# Y-Stenting for Bifurcation Aneurysm Coil Embolization: What is the Risk?

**DOI:** 10.1155/2014/762389

**Published:** 2014-07-10

**Authors:** Alejandro M. Spiotta, Jonathan Lena, M. Imran Chaudry, Raymond D. Turner, Aquilla S. Turk

**Affiliations:** ^1^Department of Neurosciences, Medical University of South Carolina, 96 Jonathan Lucas Street, CSB 428, Charleston, SC 29425, USA; ^2^Department of Radiology, Medical University of South Carolina, Charleston, SC 29425, USA

## Abstract

The use of two stents in a “Y” configuration (Y-stenting) to assist with coil embolization of complex bifurcation aneurysms has been accepted as an alternative to clip reconstruction of a select subset of challenging aneurysms. We review the risks associated with Y-stenting, including its procedural complication rates, angiographic occlusion rates, rerupture, and retreatment rates.

## 1. Introduction

Since the International Symptomatic Aneurysm Trial (ISAT) and the Barrow Ruptured Aneurysm Trial (BRAT) firmly established endovascular therapy as a valid method for treating intracranial aneurysms, development of new techniques has broadened the scope of practice to allow for the treatment of geometrically complex aneurysms. Until the introduction and widespread adoption of adjuncts to endovascular coil embolization, complex wide-necked bifurcation aneurysms had classically been treated with microsurgical clip reconstruction. Advances in endovascular techniques including balloon remodeling as well as the use of stents have allowed more of these challenging aneurysms to be treated with coil embolization. The use of two stents in a “Y” configuration (Y-stenting) to assist with coil embolization of complex bifurcation aneurysms was first described by Chow et al. in 2004 [1]. Since that time many reports have been published demonstrating low morbidity and mortality rates associated with Y-stenting [1–14] and it has been accepted as a safe and reasonable alternative to clip reconstruction of a select subset of challenging aneurysms. But what exactly are the risks associated with Y-stenting? To address this question a thorough understanding of the technical aspects of the procedure as well as the available reported rates of complications is required.

## 2. Stent Assisted Coiling

The technique of stent assisted coiling in the clinical setting was first described in 1997 [15] Soon after, the availability of new flexible, self-expanding intracranial stents allowed for increasing application of this technique and observation of its benefits. Stents have been quickly adopted as promising adjuncts with potential mechanical, hemodynamic, and biologic properties, imparting an advantage over coil embolization alone [15]. Stent deployment provides mechanical support to prevent coil prolapse, may serve as a conduit to divert flow, and provides a scaffold for endothelial growth and vessel healing [15–17]. In addition, an implanted stent may incur subtle changes in the parent vessel-aneurysm geometry, imparting significant hemodynamic alterations which change the inflow substantially and which may contribute to progressive thrombosis of even incompletely occluded aneurysms.

## 3. Y-Stent Reconstruction

Despite advances in stenting and balloon remodeling techniques, broad-necked aneurysms arising at bifurcations that incorporate the daughter vessel(s) origins remain a challenge to endovascular treatment. This is perhaps best exemplified by the difficulty in treating middle cerebral artery bifurcation aneurysms, but also those at the basilar apex and carotid terminus. At vascular bifurcations, a stent can be used to stabilize a coil mass within an aneurysm while protecting the parent vessel and the daughter vessel at greatest risk. When the aneurysm neck incorporates the origins of both daughter vessels and there is a significant risk of coil herniation into either vessel, a single stent, even with balloon remodeling as an adjunct, may not be sufficient to protect the parent vessel.

The “Y-stent” technique involves the passage of a second stent through the interstices of the first deployed stent. For example, for basilar apex aneurysms, the distal end of the first stent is positioned into the posterior cerebral artery (PCA) that arises at the most acute angle from the basilar apex (or the one with the most difficult configuration to navigate), with the proximal end positioned in the distal basilar artery itself. The second stent is navigated through the interstices of the initial stent with the distal end in the contralateral PCA and the proximal half within and overlapping the first stent in the basilar artery (“Y”), thus reconstructing the neck. The microcatheter is then navigated through the proximal true lumen of the stents and exited through the interstices into the aneurysm to achieve coil embolization (“coil through”) [18]. Of note, a variation of this technique can involve selecting the aneurysm first with a microcatheter, then depositing the stents across the aneurysm (“jailing”) (Figure 1).

## 4. Technical Considerations

Thus, Y-stent reconstruction enables the endovascular management of otherwise complex, wide-necked cerebral aneurysms by providing two critical functions: support for the coil mass and preservation of the daughter vessels. Y-stent reconstruction requires platelet inhibition to prevent in-stent thrombus formation, thromboembolic events, and vessel occlusion. Centers with high volume experience with Y-stent reconstruction recommend that dual antiplatelet inhibition with aspirin and clopidogrel is required between three and six months following the procedure. Aspirin is generally continued lifelong. Some centers pretreat with dual agents for seven to ten days preprocedure while others, like ours, administer loading doses the evening prior. Some centers routinely test platelet inhibition with laboratory testing while others do not. Importantly, no data supports the use of one strategy over another and this area remains controversial.

The open-cell design of the Neuroform stent was favored in the first descriptions of this technique [1, 11–13] and it was reasoned that the Y-stent construct was possible because the first stent deployed can expand at its interstices to accommodate the second self-expanding stent. Y-stenting using a closed-cell design stent as the initial stent (open-closed) is also technically feasible, although it was not adopted enthusiastically due to what would prove to be unfounded concerns. Theoretically, employing a closed-cell design stent as the initial stent results in undesirable synching of the second deployed stent because of its constrained interstices. Furthermore, closed-closed constructs have also been reported with success [6].

Navigating through the first stent with either an 021 delivery microcatheter (closed-cell stent) or an 028 delivery microcatheter (open-cell stent) to deploy the second stent of the construct can be challenging and result in stent migration. It is for this reason that the most difficult PCA to access is stented first. However, despite adopting this strategy, we have abandoned planned open-open Y constructs for an open-closed construct after encountering difficulties in placing an 028 microcatheter through the first stent. In these rare instances, we have found an 021 microcatheter to travel more readily and make completion of the construct, and treatment of the aneurysm, possible.

Initial microcatheter selection and reaccessing of the aneurysm during coiling following Y-stenting can also pose a challenge. In fact, there are many steps required to successfully achieve Y-stent reconstruction, each with possibility of technical complications.

## 5. Angiographic Occlusion Rates

The reported rates of immediate angiographic occlusion following Y-stent reconstruction are variable, Raymond I (26.3–43%), Raymond II (26.3–41%), and Raymond III (11.1–45.3%) [6, 12]. While the rate of residual aneurysm neck filling may appear unacceptable at first glance, it should be reminded that these aneurysms represent a small subset of basilar apex aneurysms that are exceedingly complex to address. Surgical morbidity would be expected to be high in this cohort due to the same concerns surrounding daughter vessel protection and complete aneurysm occlusion with coiling of these lesions, but with additional considerations surrounding perforator vessel preservation during neck and dome dissection, as well as clip application. Importantly, while many of the aneurysms displayed residual filling at initial treatment, some were found to have spontaneous thrombosis on angiographic follow-up [12] while the highest predictor of long-term occlusion was complete occlusion at initial treatment.

## 6. Complications

Despite inherent technical challenges, the centers reporting their experience with Y-stent reconstruction have demonstrated high technical success ranging from 88.9 to 100%, while the incidence of complications on the initial treatment remained low from 0 to 21% [6]. Rates of technical difficulties such as stent migration (3.1%), stent prolapse (3.1%), and coil herniation (1.6%) remain low combining the two largest center experiences [6, 12]. The periprocedural symptomatic ischemic stroke rate was found to be 4.7%. Thromboembolic rates range from 0 to 11.1% [6], with the highest reported rates reported in the early experience [12]. This difference may reflect a broader acceptance of more long-term dual antiplatelet therapy based on the preliminary data, specifically a reluctance to discontinue clopidogrel prior to six months. The incidence of delayed strokes (greater than two weeks after procedure) is low at 3.1% [6, 12]. There have been no reports of in-stent stenosis (7.8% incidence) requiring treatment. All studies report a zero procedural mortality rate.

Aneurysm recurrence rates range from 8.9 to 28.6% at from 3.5 to 36.7% months follow-up. Retreatment rates range from 0–15% [6, 12].

## 7. Conclusions

Wide-necked bifurcation aneurysms represent a difficult subset of aneurysms to treat. These aneurysms occur at the junction of two essential branching arteries that must remain patent after embolization. Unassisted coiling or single stent techniques are frequently insufficient to protect the remaining daughter vessels and prevent coil prolapse leading to arterial occlusion. Flow-diversion stenting is not ideal because this would effectively jail one of the limbs of the bifurcation.

Despite the technical challenges associated with stent assisted coil embolization of bifurcation aneurysms using a Y configuration, this technique appears to be safe with low morbidity and mortality. Thus, Y-stent coiling appears to be the best treatment option for these challenging aneurysms.

## Figures and Tables

**Figure 1:**